# Predictive model of the first failure pattern in patients receiving definitive chemoradiotherapy for inoperable locally advanced non-small cell lung cancer (LA-NSCLC)

**DOI:** 10.1186/s13014-020-1467-x

**Published:** 2020-02-18

**Authors:** Xueru Zhu, Runping Hou, Xiaoyang Li, Chang Jiang, Wuyan Xia, Xiaolong Fu

**Affiliations:** 1 Department of Radiation Oncology, Shanghai Chest Hospital, Shanghai Jiao Tong University, Shanghai, 200030 China; 2 Department of Biomedical Engineering, School of Biomedical Engineering, Shanghai Jiao Tong University, Shanghai, 200030 China

**Keywords:** Locally advanced non-small cell lung cancer, Failure, Recurrence, Predictor

## Abstract

**Purpose:**

To analyze patterns of failure in patients with LA-NSCLC who received definitive chemoradiotherapy (CRT) and to build a nomogram for predicting the failure patterns in this population of patients.

**Materials and methods:**

Clinicopathological data of patients with LA-NSCLC who received definitive chemoradiotherapy and follow-up between 2013 and 2016 in our hospital were collected. The endpoint was the first failure after definitive chemoradiotherapy. With using elastic net regression and 5-fold nested cross-validation, the optimal model with better generalization ability was selected. Based on the selected model and corresponding features, a nomogram prediction model was built. This model was also validated by ROC curves, calibration curve and decision curve analysis (DCA).

**Results:**

With a median follow-up of 28 months, 100 patients experienced failure. There were 46 and 54 patients who experience local failure and distant failure, respectively. Predictive model including 9 factors (smoking, pathology, location, EGFR mutation, age, tumor diameter, clinical N stage, consolidation chemotherapy and radiation dose) was finally built with the best performance. The average area under the ROC curve (AUC) with 5-fold nested cross-validation was 0.719, which was better than any factors alone. The calibration curve revealed a satisfactory consistency between the predicted distant failure rates and the actual observations. DCA showed most of the threshold probabilities in this model were with good net benefits.

**Conclusion:**

Clinicopathological factors could collaboratively predict failure patterns in patients with LA-NSCLC who are receiving definitive chemoradiotherapy. A nomogram was built and validated based on these factors, showing a potential predictive value in clinical practice.

## Introduction

According to the latest NCCN guidelines, definitive chemoradiotherapy is the main recommended treatment for unresectable LA-NSCLC [1]. Due to the excellent result of the PACIFIC trial [2], an immune checkpoint inhibitor (ICI) was recommended for subsequent maintenance therapy. Patients with this disease staging were heterogeneous, and the 5-year survival rates ranged from 6 to 30% [3]. A previous study showed that radiation technology (intensity modulated radiation therapy (IMRT) vs three-dimensional conformal radiotherapy (3D-CRT)) may be the predictive factor of recurrence patterns [4]. However, nearly all patients receiving definitive radiotherapy in our institution were treated by IMRT, and the failure patterns of these patients were different from those previously described.

Concurrent chemoradiotherapy (CCRT) improved the survival of patients with LA-NSCLC compared with sequential chemoradiotherapy (SCRT), indicating that local control is important for patient outcome [5]. Obtaining good locoregional control is the main goal in LA-NSCLC. Studies have shown that improving radiation doses or performing large segmentation may improve local control, but doses to the surrounding organs at risk were also increased [6–8]. Therefore, clearly classifying the failure patterns among these patients is the basis for precise radiation treatment. Currently, no satisfactory predictive model has been built to assess the failure patterns for LA-NSCLC.

Here, we present the results of a retrospective study analyzing the failure patterns in 100 patients with LA-NSCLC who received definitive CRT. The purpose of this study was to investigate the first failure pattern of LA-NSCLC and build a nomogram to predict the failure pattern for these patients.

## Materials and methods

### Patients

Between 2013 and 2016, consecutive patients underwent definitive CRT for LA-NSCLC and were followed-up in our hospital. The inclusion criteria were as follows: (1) patients initially diagnosed with locally advanced lung cancer; (2) patients with pathologically confirmed non-small cell lung cancer; (3) patients who received IMRT to unresectable LA-NSCLC; (4) patients with a total radiation dose ≥50 Gy and who had completed the radiotherapy plan; (5) patients with data on the determination of EGFR mutation status; (6) patients who received regular follow-up imaging examinations (thoracic CT, brain MRI, abdominal and superficial lymph node ultrasound, whole-body bone scan or PET-CT) in our hospital. The exclusion criteria were as follows: (1) patients with a second primary cancer; (2) patients who died during the course of radiotherapy; (3) patients with an unknown EGFR status; (4) patients who were treated with maintenance chemotherapy or targeted therapy after definitive CRT; (5) patients who received targeted therapy before the first failure; and (6) patients with unavailable clinicopathological materials. Finally, 100 patients were included in this study.

### Definition of clinicopathological factors and recurrence/metastasis

Data on clinicopathological factors, including age, gender, family history, chronic medical diseases, smoking status, pathological diagnosis, chemotherapy, EGFR mutation status, TNM stage (according to the TNM classification in the Union for International Cancer Control (UICC) 8th ed. [9]), the radiotherapy and chemotherapy regimens delivered, and radiotherapy planning margins were retrieved. Imaging examination findings, including those from thoracic CT, brain MRI, abdominal and superficial lymph node ultrasound, whole-body bone scan or PET-CT before and after treatments, were reviewed to evaluate the clinical stage and failure patterns. Disease recurrence at the in-field (centroid originating within the original planning target volume) of radiation or local-regional lymph node was considered local failure, and all other sites of recurrence or metastases were defined as distant failure. Failure patterns were evaluated by imaging examinations, and the first sites of recurrence were recorded.

### Statistical analysis

Baseline characteristics are shown as numbers. Univariate analysis with Chi-square test and Wilcoxon test were conducted to explore individual significant factors with false discovery rate (FDR) correction. The model building process were divided into two steps. First, based on all the 16 clinical factors, multivariate logistic regression with elastic net regularization was used to generate models including different features. In this stage, model training and parameter tuning were based on the whole dataset. Second, 5-fold nested cross-validation was conducted to assess the generalization ability of those models incorporating different features. Finally, the selected optimal model and the corresponding features were used to build a nomogram. The AUC of the ROC curve, calibration curve and DCA were also assessed to validate the performance of the predictive model. The dataset and scripts were attached as supplement material.

Statistical analysis was performed using SPSS software, version 22.0, R software, version 3.4.5 and python version 3.7. All tests were two-sided, and *p* < 0.05 was defined as a statistically significant result.

## Results

### Patient characteristics

The baseline characteristics of the included LA-NSCLC patients are shown in Table 1. Of the 100 patients, the median age was 59 (22–83) years old. Eighty patients were males, and 20 were females. There were 38, 56 and 6 patients diagnosed with clinical stages of IIIA, IIIB and IIIC, respectively. The tumor location was peripheral in 59 patients, and the other 41 patients’ lesions were located centrally. Forty-nine patients were pathologically diagnosed with adenocarcinoma, 39 with squamous cell carcinoma and 12 with non-small cell carcinoma. Among the 100 patients, EGFR mutations were detected in 24. All patients were treated with IMRT technology, 79% received a radiation dose of 60 Gy/30 Fx, and the median radiation dose was 60 Gy (50–66 Gy). Forty-three patients who were treated with 2–4 cycles of consolidation chemotherapy after radiation. Only 40 patients were given CCRT, and 60 patients were treated with SCRT.

**Table 1:** Patient characteristics

Characteristic	Data (*n* = 100)	Characteristic	Data (*n* = 100)
Age, y	Median, 59 (22–83)	EGFR mutation status	
Sex		Wild	76
Female	20	Mutant	24
Male	80	Clinical T stage	
Smoking		cT1	31
No	40	cT2	33
Yes	60	cT3	20
Pathology		cT4	16
Non-small cell carcinoma	12	Clinical N stage	
Adenocarcinoma	49	cN0–1	7
Squamous cell carcinoma	39	cN2	53
Tumor diameter		cN3	40
≤3 cm	35	Sequence of CRT	
> 3 cm, ≤5 cm	36	Concurrent	40
> 5 cm, ≤7 cm	19	Sequential	60
> 7 cm	10	Consolidation chemotherapy chemotherapy	
Lung lobe		No	57
Upper and middle	74	Yes	43
Lower	26	Radiation Dose	
Location		< 60 Gy	14
Central	41	≥60 Gy	86
Peripheral	59	Primary tumor volume (cm^3^)	Median, 45.19(0.89–392.9)
Clinical TNM stage		Lymph Nodal volume (cm^3^)	Median, 63.7(0–335.86)
cIIIA	36	Total volume (cm^3^)	Median, 140.0(19.79–481.28)
cIIIB	58		
cIIIC	6		

*CRT* chemoradiotherapy

**Fig. 1:**
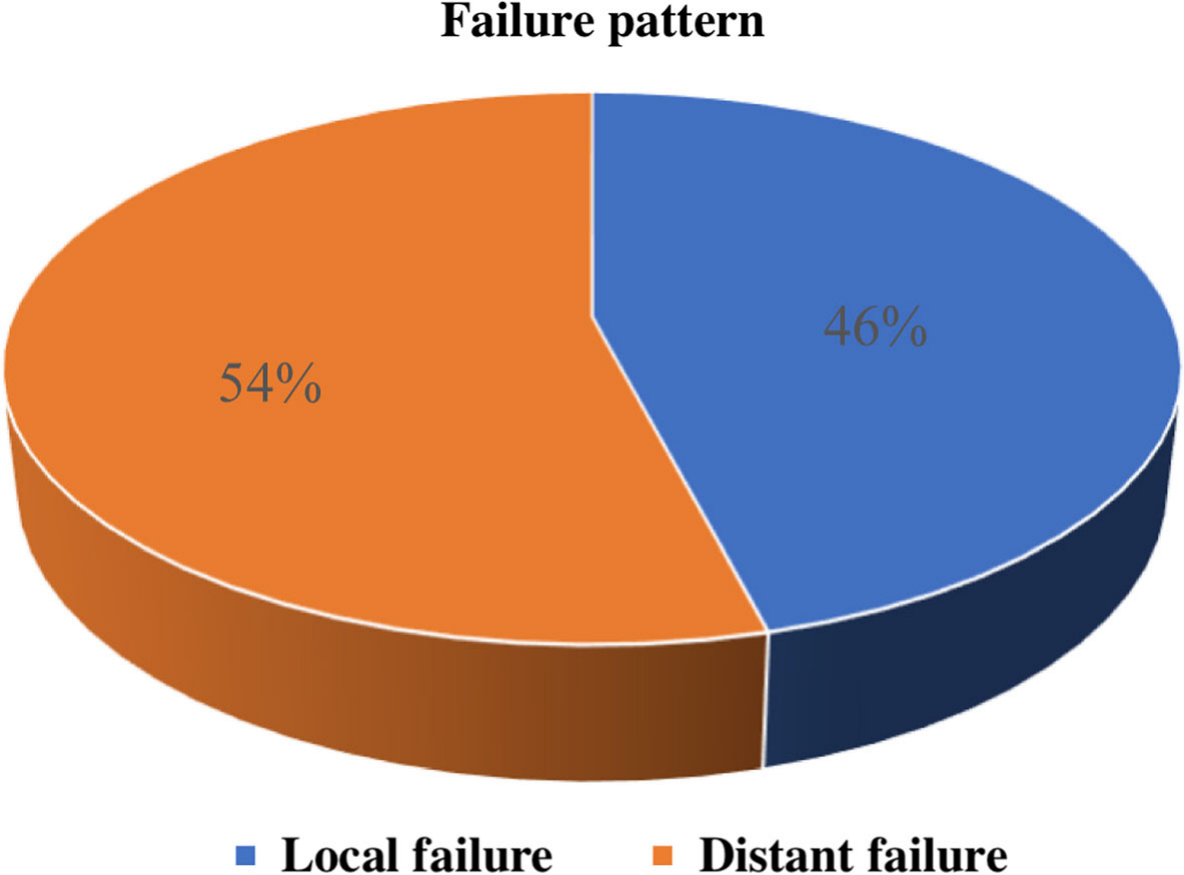
The distribution of first failure patterns among patients with inoperable locally advanced non-small cell lung cancer who received definitive chemoradiotherapy

With a median follow-up of 28 months, among the 100 patients who exhibited failure, 46 and 54 experienced local failure and distant failure, respectively (Fig. 1).

### Univariate analysis

Univariate analysis with Chi-square test (categorical predictors) and Wilcoxon test (continuous predictors) showed that sex (*p* = 0.035), smoking status (*p* = 0.027), pathological tumor type (*p* = 0.031), age (*p* = 0.026), tumor location (*p* = 0.012) and EGFR mutation status (*p* = 0.018) were statistically associated with the different failure patterns. However, considering the multiple comparison, FDR correction was conducted and the adjusted q values were shown in Table 2.

**Table 2:** Univariate analysis of clinical factors in predicting failure pattern

Characteristic	Local failure (*n* = 46)	Distant failure (*n* = 54)	Chi-square test *P* value	FDR correction *Q* value
Sex			**0.035**	0.093
Female	5 (25.0%)	15 (75.0%)		
Male	41 (51.2%)	39 (48.8%)		
Smoking			**0.027**	0.093
No	13 (32.5%)	27 (67.5%)		
Yes	33 (55.0%)	27 (45.0%)		
Pathology			**0.031**	0.093
Squamous cell carcinoma	24 (61.5%)	15 (38.5%)		
Non-small cell carcinoma	3 (25.0%)	9 (75.0%)		
Adenocarcinoma	19 (38.8%)	30 (61.2%)		
Tumor diameter			0.284	0.432
≤3 cm	13 (37.1%)	22 (62.9%)		
> 3 cm, ≤5 cm	20 (55.6%)	16 (44.4%)		
> 5 cm, ≤7 cm	10 (52.6%)	9 (47.4%)		
> 7 cm	3 (30.0%)	7 (70.0%)		
Location			**0.012**	0.093
Central	25 (61.0%)	16 (39.0%)		
Peripheral	21 (35.6%)	38 (64.4%)		
EGFR			**0.018**	0.093
Wild	40 (52.6%)	36 (47.4%)		
Mutant	6 (25%)	18 (75%)		
Clinical T stage			0.260	0.432
cT1	10 (32.3%)	21 (67.7%)		
cT2	18 (54.5%)	15 (45.5%)		
cT3	11 (55.0%)	9 (45.0%)		
cT4	7 (43.8%)	9 (56.3%)		
Clinical N stage			0.297	0.432
cN0–1	5 (71.4%)	2 (28.6%)		
cN2	25 (47.2%)	28 (52.8%)		
cN3	16 (40.0%)	24 (60.0%)		
Clinical TNM stage			0.267	0.432
cIIIA	19 (52.8%)	17 (47.2%)		
cIIIB	23 (39.7%)	35 (60.3%)		
cIIIC	4 (66.7%)	2 (33.3%)		
Sequence of CRT			0.566	0.604
Concurrent	17(37.0%)	29(63.0%)		
Sequential	23(42.6%)	31(57.4%)		
Consolidation chemotherapy			0.087	0.199
No	22(38.6%)	35(61.4%)		
Yes	24(55.8%)	19(44.2%)		
Radiation Dose			0.405	0.498
< 60 Gy	5 (35.7%)	9 (64.3%)		
≥60 Gy	41 (47.7%)	45 (52.3%)		
^*^Age (y) (continuous variable)	m^#^61.5(38–83)	m^#^59(22–73)	**0.026**	**0.093**
^*^Primary tumor volume (cm^3^)	m^#^47.04(5.12–383.14)	m^#^38.17(0.89–392.9)	0.377	0.498
^*^Lymph nodal volume (cm^3^)	m^#^62.68(0–241.92)	m^#^65.69(0–335.86)	0.966	0.966
^*^Total volume (cm^3^)	m^#^142.84(19.79–473.64)	m^#^129.58(27.25–481.28)	0.487	0.557

*CRT* chemoradiotherapy

^#^ For continuous variables, “m” means median, and ranges of variables are in parentheses. ^*^For these continuous variables, Wilcoxon rank-sum test was used

Due to the limitation of small sample size, we included all 16 factors which may affect the failure patterns, into the multivariate analysis of elastic net regression.

### Development and validation of the failure pattern prediction model

The tuned hyperparameter and the generated five models with different number of features in the first stage are shown in Table 3. Assessment results of the five models using 5-fold nested cross-validation is shown in Table 4. Results shown that the optimal model incorporated nine features including smoking, pathology, location, EGFR mutation status, age, tumor diameter, clinical N stage, consolidation chemotherapy and radiation dose. The detailed coefficients and corresponding hyperparameter information of the optimal model can be found in Additional file 1: Table S1.

**Table 3:** Models and corresponding features generated by elastic net regression

Features Alpha	Alpha = 0	Alpha = 0.1	Alpha = 0.2–0.3	Alpha = 0.4–0.7	Alpha = 0.8–1.0
Feature numbers	16	13	11	10	9
Features	Sex, Smoking, Pathology, Location, EGFR mutation status, Age, Tumor diameter, Clinical T stage, Clinical N stage, Clinical TNM stage, Sequence of CRT, Consolidation chemotherapy, Radiation dose, Primary tumor volume, Lymph nodal volume	Sex, Smoking, Pathology, Location, EGFR mutation status, Age, Tumor diameter, Clinical T stage, Clinical N stage, Consolidation chemotherapy, Radiation dose, Primary tumor volume, Lymph nodal volume	Sex, Smoking, Pathology, Location, EGFR mutation status, Age, Tumor diameter, Clinical N stage, Consolidation chemotherapy, Radiation dose, Primary tumor volume	Sex, Smoking, Pathology, Location, EGFR mutation status, Age, Tumor diameter, Clinical N stage, Consolidation chemotherapy, Radiation dose	Smoking, Pathology, Location, EGFR mutation status, Age, Tumor diameter, Clinical N stage, Consolidation chemotherapy, Radiation dose

**Table 4:** Model assessment by 5-fold nested cross-validation

	AUC of 5-fold nested cross-validation	Average AUC
Model with 16 features	0.719, 0.539, 0.587, 0.768, 0.748	0.672
Model with 13 features	0.750, 0.725, 0.533, 0.707, 0.747	0.692
Model with 11 features	0.750, 0.725, 0.533, 0.798, 0.747	0.709
Model with 10 features	0.750, 0.758, 0.533, 0.788, 0.747	0.715
Model with 9 features	0.739, 0.725, 0.546,0.778, 0.808	0.719

**Fig. 2:**
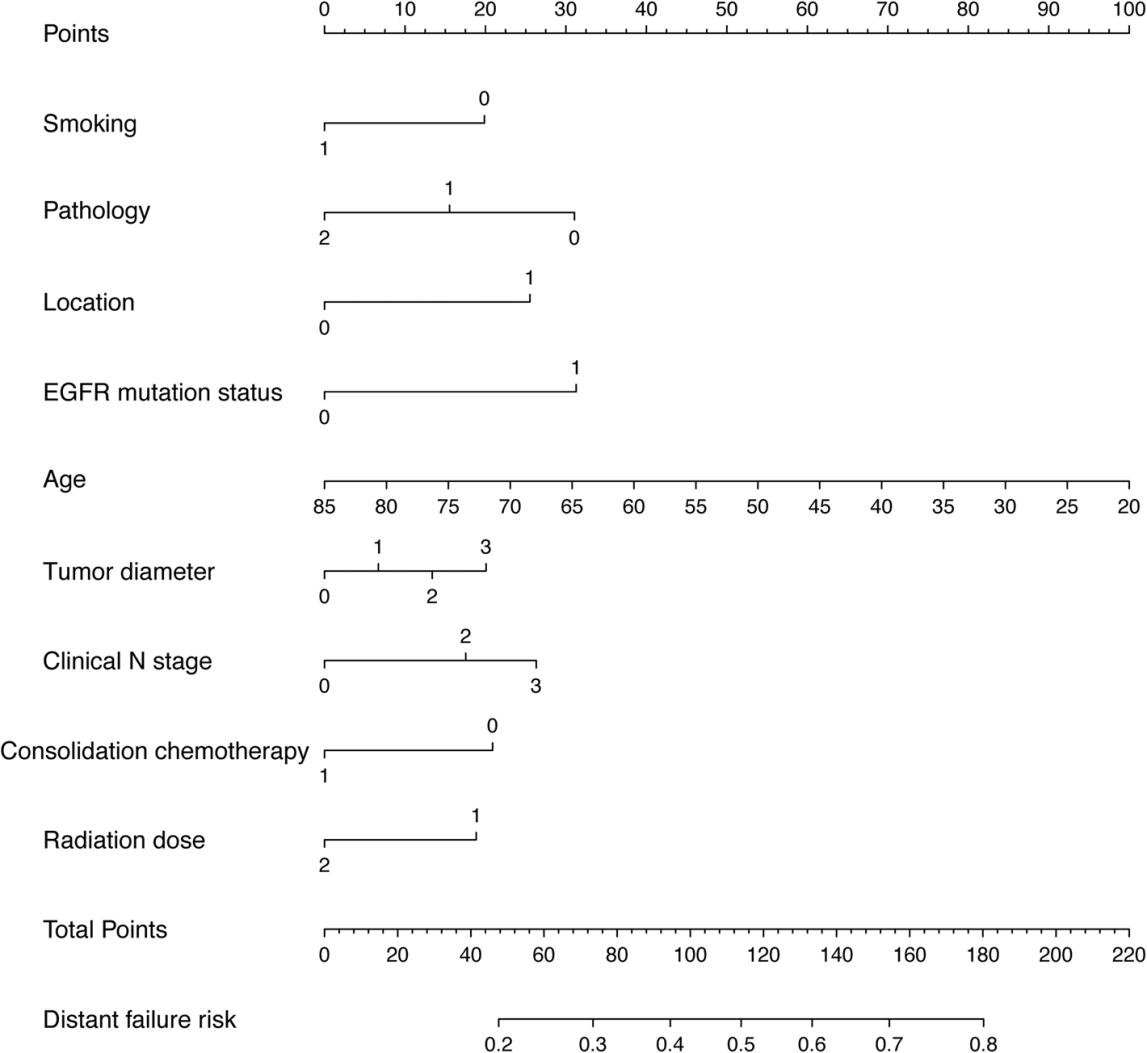
Nomogram predicting the first failure patterns. For each individual patient, nine lines are drawn upward to determine the points received from the nine variables in the nomogram. The sum of these points is located on the “Total Points” axis, and a line is drawn downward to determine the likelihood that this patient to occur distant failure

**Fig. 3:**
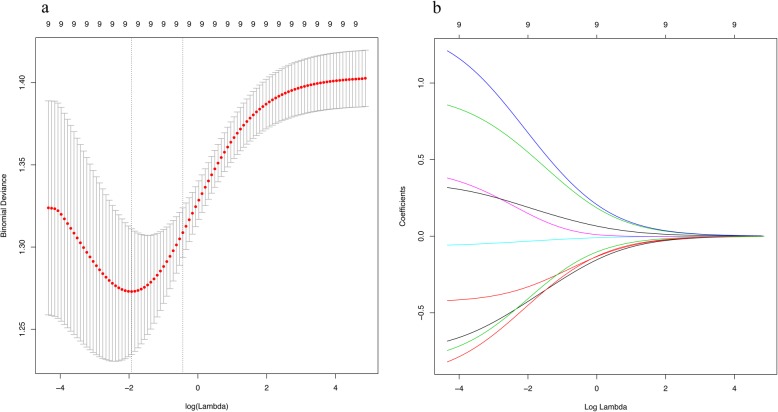
Parameter tuning of the elastic net logistic regression. **a** Selection of the tuning parameter lambda in the elastic net model via 5-fold cross validation based on minimum criteria. The x-axis represents the different log(lambda). The y-axis represents the binomial deviance. Numbers along the upper x-axis represent the average number of predictors. Red dots represent the average binomial deviance values of each model with a given lambda. The vertical bars represent the upper and lower value of the deviances. The vertical black lines represent the optimal lambda which fits the data best. **b** Elastic net regression coefficient of the nine factors with different lambda values. The x-axis and y-axis represent the log(lambda) and coefficient. Different color lines were representing different factors

**Fig. 4:**
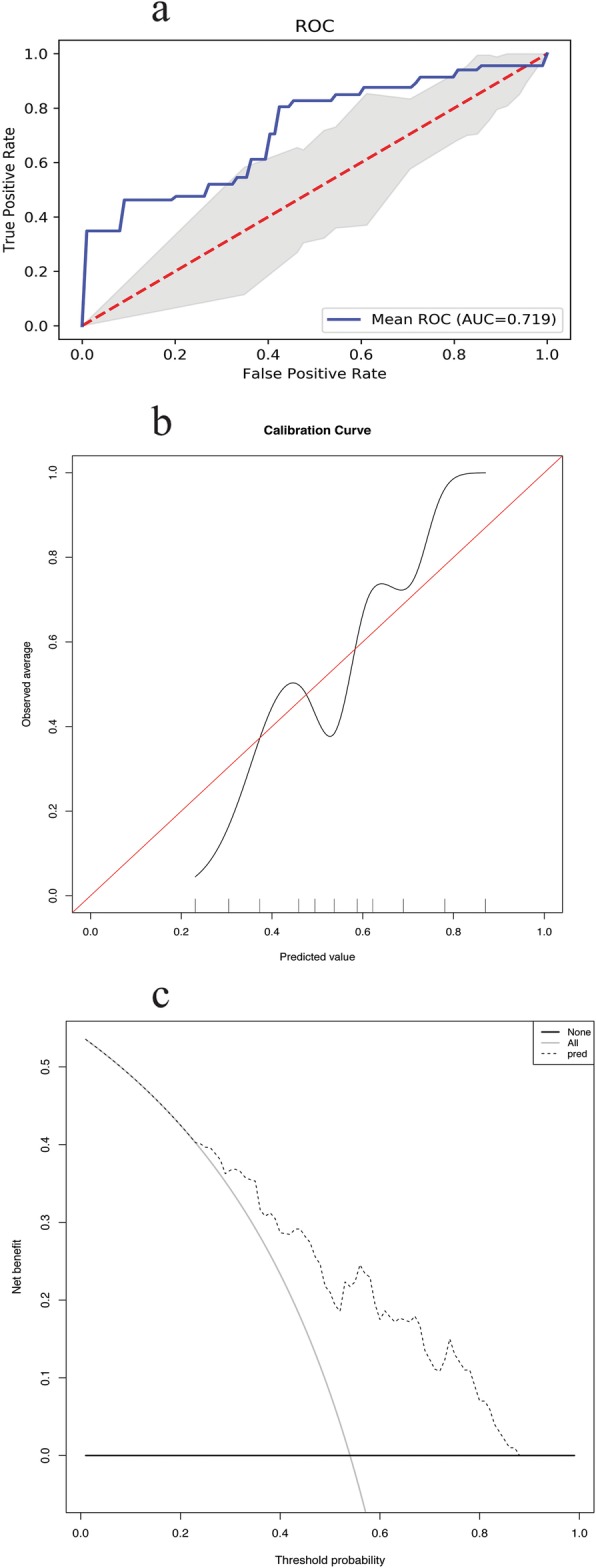
The validation of nomogram. **a** Average ROC curve of the prediction model with 5-fold nested cross-validation. The average AUC of the 5-fold validation was 0.719. **b** Calibration curve of the nomogram model to predict distant failure. The x-axis and y-axis represent the predicted and actual probabilities of occurring distant failure, respectively. The blue diagonal 45 line correspond to the perfect prediction, and the orange dotted line represent the predictive performance of the nomogram. The closer the dotted line fitted to the diagonal line, the better the prediction performance was. **c** Decision curve of the nomogram to predict distant failure in the whole dataset. The x-axis and y-axis measure the threshold probabilities and net benefit, respectively. The latter is calculated by adding the true positives and subtracting the false positives. The horizontal line along the x-axis assumes that no patient will occur distant failure, the solid gray line assumes that all patients will occur distant failure at a specific threshold probability. The solid blue line means the net benefit with using the nomogram

Based on the selected nine features, a failure pattern predictive model is visually presented as a nomogram (Fig. 2). Example of the hyperparameter tuning details can be seen from Fig. 3. In Fig. 4a, we show the average ROC curve of the predictive model with 5-fold nested cross-validation and the average AUC was 0.719 (95% CI: 0.474–0.963). The ROC curves of smoking, pathology, location, EGFR mutation status, age, tumor diameter, clinical N stage, consolidation chemotherapy, radiation dose and the nomogram indicated that the nomogram predicting model was excellent and better than each factor alone (age: AUC = 0.630, 95% CI = 0.520–0.734; tumor location: AUC = 0.624, 95% CI = 0.528–0.719; EGFR mutation status: AUC = 0.601, 95% CI = 0.521–0.682; Pathology: AUC = 0.638, 95% CI = 0.540–0.737; Smoking: AUC = 0.609, 95% CI = 0.515–0.703; Tumor diameter: AUC = 0.531, 95% CI = 0.423–0.640; clinical N stage: AUC = 0.566, 95% CI = 0.466–0.667; Consolidation chemotherapy: AUC = 0.585, 95% CI = 0.488–0.682; Radiation dose: AUC = 0.529, 95% CI = 0.461–0.597) to predict distant failure (Additional file 2: Figure S1). The results showed that for a single factor, age was the most suitable for predicting local failure (AUC =0.630, 95% CI = 0.520–0.734), while tumor location and pathology were better at predicting distant failure than at predicting local failure. The nomogram showed good predictive efficiency (specificity 81.8%, sensitivity 69.4%). The validation results of the calibration curve showed satisfactory consistency between the predicted distant failure and actual observation (Fig. 4b). DCA showed that the majority of the threshold probabilities in this model had good net benefits (Fig. 4c).

## Discussion

For stage III NSCLC, which is a heterogeneous disease, a uniform standardized treatment is inadequate, and an individualized precise treatment is needed. Before precise treatment can be provided, a clear classification of patients with this stage is fundamental. This study was conducted to build a nomogram for predicting the failure pattern in LA-NSCLC patients. Studies have shown that the most common type of recurrence is distant [10–12], which is consistent with our cohort (distant failure vs. local failure = 54% vs. 46%). Previous reports have suggested that the presence of EGFR mutation is a favorable factor for local control, and patients with EGFR mutation were at a high risk of distant relapse [13–17]. Considering the significant role of EGFR mutation status in predicting failure pattern, we only included LA-NSCLC patients who detected the EGFR mutation status in this study.

Previous studies have indicated that the prevalence of EGFR mutations in stage III NSCLC patients ranges from 17 to 30%, which was similar to that in our cohort (24%) [14, 16, 17]. Consistent with the results in previous studies, our study also showed that EGFR mutant patients of LA-NSCLC who are receiving definitive chemoradiotherapy have a higher distant failure rate than a local failure rate. However, the predictive efficacy of only one factor was not satisfactory, and we need a combined and high-efficacy predictive model. Hence, in this study, we included 16 clinicopathological factors that were considered to be related to the recurrence or patient prognosis to build a nomogram model [18–21]. In the univariate analysis, we observed 6 factors that may be related to the failure patterns. The 6 factors were sex, smoking status, pathological type, age, tumor location and EGFR mutation status. Considering the multiple comparison of univariate analysis, we corrected it with FDR. After the FDR correction, above 6 factors still showed the predictive trend for failure pattern.

However, since univariate analysis does not consider interactions among features, we directly utilized a multivariate elastic net regression of these 16 clinical features without filtering features using univariate analysis. Elastic net is a hybrid of Lasso and Ridge regression techniques by using L1 and L2 norms as priori regularization during training. The hyperparameter alpha was used to control the convex combination of L1 and L2 regularization. Consequently, this combination enables the elastic net select features intrinsically. Here, the elastic net (with 5-fold nested cross-validation) finally selected nine features including smoking, pathology, location, EGFR mutation status, age, tumor diameter, clinical N stage, consolidation chemotherapy and radiation dose to build prediction model. The statistical results indicated that LA-NSCLC patients receiving definitive chemoradiotherapy with the characteristics of non-smoker, non-squamous cell carcinoma, younger age, peripheral LA-NSCLC, EGFR mutations, long tumor diameter, advanced N stage, non-consolidation chemotherapy and low radiation dose were more likely to experience distant failure. To date, few studies have compared the failure pattern between peripheral and central LA-NSCLC.

In previous study, Brian et al. [22] reported that only TNM stage and surgery but not primary tumor or lymph node volume were associated with distant failure. In our study, we also did not observe a relationship between tumor volume or lymph nodal volumes with distant failure. In our cohort, the predictive role of clinical N stage for failure pattern was confirmed. In a poster report shown at the American Society for Radiation Oncology (ASTRO) annual meeting 2016, Yoon et al. [4] showed that the use of IMRT decrease the risks of locoregional failure and adjuvant chemotherapy correlated with a decrease in distant failure. In our study, all patients were treated with IMRT. All patients received chemotherapy and radiotherapy, 40 patients were given CCRT, and 60 patients were treated with SCRT. Among the 100 patients, 43 patients were treated with 2–4 cycles of consolidation chemotherapy after radiation. Study result also showed that whether conducted consolidation chemotherapy was related to the failure pattern. Otherwise, in a study of early NSCLC, Masaki et al. [23] reported that patients with early-stage peripheral NSCLC were more likely to have distant failure. In a study by Melisa et al. [24], their data indicated that the recurrence rate of distant sites was higher than that at local sites in younger patients with NSCLC and peripheral NSCLC. Although the populations were different between our cohort and the above two studies, we obtained similar predictors for the first failure pattern for NSCLC patients. Furthermore, we found that the driver gene mutation status of NSCLC was associated with distant failure. A recent study also indicated that compared to patients with wild-type EGFR stage III NSCLC, those with EGFR-mutant unresectable stage III NSCLC tend to have a higher rate of out-of-field failure than in-field failure [25]. However, in the majority of the previous studies mentioned above, no predictive model was developed for a single impact factor. Hence, we built a nomogram to improve the predictive power of failure patterns for these stage III patients.

Different rates of local recurrence and distant failure according to the predictive model provide significant insights into the therapeutic strategies for LA-NSCLC. To improve the local control rate in patients with unresectable LA-NSCLC, several dose-escalation studies were conducted [6, 26, 27]. The classic study of RTOG 0617 showed that high dose (74 Gy) radiation did not result in a better outcome than the outcome of the conventional dose (60 Gy). The main reason for the poor outcome in the high-dose group may be that the treatment-related adverse event rate was higher in the high-dose group than in the conventional-dose group. In patients who tended to experience distant failure of the first recurrence, high-dose radiation only increased the radiation-related side effects rather than increasing the benefit of local control. Therefore, fitting populations are significant and necessary for a rigorous study, and accurate classification of the population is fundamental to providing precise treatment. The data also indicated that the outcomes between local failure and distant failure were different [16, 25, 26]. Hence, to design a better treatment strategy, we should make a good prediction of failure patterns before beginning treatment.

Certainly, the limitations of our study should be addressed here. The sample size of this study (*n* = 100) is relatively small. This is a retrospective analysis, and large sample sizes and rigorous prospective studies are needed to validate this predictive model. Moreover, the sensitivity of this model to predict distant failure was poor, but the specificity was excellent. Although sensitivity and specificity were both important, for this study, the sensitivity may not be as important as the specificity, because the whole precision of the model is compromised. Furthermore, the calibration curve revealed a good consistency between the predicted distant failure and actual observation. Certainly, an external validation was needed, which will make the predictive model more convincing. In addition, the dimension of our data was low, more features including clinicopathological factors, imaging features, biological features and so on, were needed to make a standardized predictive model. Even so, all of these cases were from the real world and this predictive model can still provide good assistance in clinical practice.

## Conclusion

This study discovered collaborating clinicopathological factors could predict the first failure pattern in patients with LA-NSCLC who are receiving definitive chemoradiotherapy. The predictive model of nomogram built by these factors (smoking, pathology, location, EGFR mutation status, age, tumor diameter, clinical N stage, consolidation chemotherapy and radiation dose) shows a potential predictive value in clinical practice.

## Supplementary information

**Additional file 1: Table S1.** Model coefficients and hyperparameters of the prediction model with 5-fold nested cross-validation.

**Additional file 2: Figure S1.** The ROC curve of individual factors including smoking, pathology, location, EGFR mutation status, age, tumor diameter, clinical N stage, consolidation chemotherapy and radiation dose to predict the distant failure among patients with inoperable local advanced non-small cell lung cancer receiving definitive chemoradiotherapy.

## Data Availability

Please contact author for data requests.

## Abbreviations

3D-CRT: Three-dimensional conformal radiotherapy
ASTRO: American Society for Radiation Oncology
AUC: Area under the ROC curve
CCRT: Concurrent chemoradiotherapy
CRT: Chemoradiotherapy
DCA: Decision curve analysis
EGFR: Epidermal growth factor receptor
FDR: False discovery rate
ICI: Immune checkpoint inhibitor
IMRT: Intensity modulated radiation therapy
LA-NSCLC: Locally advanced non-small cell lung cancer
SCRT: Sequential chemoradiotherapy
