# Supplementary material for: Predictive model of the first failure pattern in patients receiving definitive chemoradiotherapy for inoperable locally advanced non-small cell lung cancer (LA-NSCLC)
Source: Radiat Oncol. 2020 Feb 18;15:43. doi: 10.1186/s13014-020-1467-x (PMC7029470; doi:10.1186/s13014-020-1467-x)
Supplement: Supplementary file 1 — Additional file 1: Table S1. Model coefficients and hyperparameters of the prediction model with 5-fold nested cross-validation. [file 13014_2020_1467_MOESM1_ESM.docx]

**Supplement table 1. Model coefficients and hyperparameters of the prediction model with 5-fold nested cross-validation.**

|  | **Fold1** | **Fold2** | **Fold3** | **Fold4** | **Fold5** | **Whole** |
| --- | --- | --- | --- | --- | --- | --- |
| Smoke | 0.317 | -0.067 | -0.458 | -0.366 | -0.337 | -0.416 |
| Pathology | -0.445 | -0.332 | -0.307 | -0.109 | -0.327 | -0.325 |
| Location | 0.413 | 0.414 | 0.415 | 0.409 | 0.468 | 0.534 |
| EGFR | 0.834 | 0.258 | 0.636 | 0.365 | 0.521 | 0.654 |
| Age | -0.042 | -0.009 | -0.059 | -0.018 | -0.022 | -0.032 |
| Size | 0.255 | 0 | 0.063 | 0.021 | 0.086 | 0.140 |
| N stage | 0.181 | 0 | 0.282 | 0.120 | 0.153 | 0.183 |
| Consolidation | -0.521 | -0.043 | -0.824 | -0.226 | -0.238 | -0.437 |
| Dose | -0.465 | 0 | -0.032 | -0.308 | -0.344 | -0.394 |
| alpha  lambda | 0  0.125 | 0.3  0.164 | 0.2  0.080 | 0  0.367 | 0  0.239 | 0  0.146 |
